# Correction to: Alzheimer’s genetic risk factor FERMT2 (Kindlin-2) controls axonal growth and synaptic plasticity in an APP-dependent manner

**DOI:** 10.1038/s41380-020-01015-8

**Published:** 2021-01-04

**Authors:** Fanny Eysert, Audrey Coulon, Emmanuelle Boscher, Anaїs-Camille Vreulx, Amandine Flaig, Tiago Mendes, Sandrine Hughes, Benjamin Grenier-Boley, Xavier Hanoulle, Florie Demiautte, Charlotte Bauer, Mikael Marttinen, Mari Takalo, Philippe Amouyel, Shruti Desai, Ian Pike, Mikko Hiltunen, Frédéric Chécler, Mélissa Farinelli, Charlotte Delay, Nicolas Malmanche, Sébastien S. Hébert, Julie Dumont, Devrim Kilinc, Jean-Charles Lambert, Julien Chapuis

**Affiliations:** 1 Universite de Lille, Inserm, CHU Lille, Institut Pasteur de Lille, U1167—RID-AGE—Facteurs de Risque et Determinants Moleculaires des Maladies Liees au Vieillissement, 59019 Lille, France; 2 Centre de Recherche du CHU de Quebec-Universite Laval, CHUL, Axe Neurosciences, Quebec City, QC Canada; 3 Faculte de Medecine, Departement de Psychiatrie et de Neurosciences, Universite Laval, Quebec City, QC Canada; 4 E-Phy-Science, Bioparc de Sophia Antipolis, 2400 route des Colles, 06410 Biot, France; 5 Universite de Lille, CNRS, UMR8576—Labex DISTALZ, 59655 Villeneuve d’Ascq, France; 6 Universite Cote d’Azur, Inserm, CNRS, IPMC, DistAlz Laboratory of Excellence, Valbonne, France; 7 Institute of Biomedicine, University of Eastern Finland, Kuopio, Finland; 8 Proteome Sciences plc, Hamilton House, London, WC1H 9BB UK

**Keywords:** Diseases, Neuroscience, Molecular biology, Genetics

Correction to: *Molecular Psychiatry* (2020)

10.1038/s41380-020-00926-w

Article published online 03 November 2020

The article *Alzheimer’s genetic risk factor FERMT2 (Kindlin-2) controls axonal growth and synaptic plasticity in an APP-dependent manner*, written by Fanny Eysert, Audrey Coulon, Emmanuelle Boscher, Anaїs-Camille Vreulx, Amandine Flaig, Tiago Mendes, Sandrine Hughes, Benjamin Grenier-Boley, Xavier Hanoulle, Florie Demiautte, Charlotte Bauer, Mikael Marttinen, Mari Takalo, Philippe Amouyel, Shruti Desai, Ian Pike, Mikko Hiltunen, Frédéric Chécler, Mélissa Farinelli, Charlotte Delay, Nicolas Malmanche, Sébastien S. Hébert, Julie Dumont, Devrim Kilinc, Jean-Charles Lambert, and Julien Chapuis, was originally published electronically on the publisher’s internet portal on 3 November 2020 without open access. With the author(s)’ decision to opt for Open Choice the copyright of the article changed on 6 October 2020 to © The Author(s) 2020 and the article is forthwith distributed under the terms of the Creative Commons Attribution 4.0 International License, which permits use, sharing, adaptation, distribution and reproduction in any medium or format, as long as you give appropriate credit to the original author(s) and the source, provide a link to the Creative Commons licence, and indicate if changes were made. The images or other third party material in this article are included in the article’s Creative Commons licence, unless indicated otherwise in a credit line to the material. If material is not included in the article’s Creative Commons licence and your intended use is not permitted by statutory regulation or exceeds the permitted use, you will need to obtain permission directly from the copyright holder. To view a copy of this licence, visit http://creativecommons.org/licenses/by/4.0.

